# The Role of Imaging in the Detection of Non-COVID-19 Pathologies during the Massive Screening of the First Pandemic Wave

**DOI:** 10.3390/diagnostics12071567

**Published:** 2022-06-28

**Authors:** Perrine Canivet, Colin Desir, Marie Thys, Monique Henket, Anne-Noëlle Frix, Benoit Ernst, Sean Walsh, Mariaelena Occhipinti, Wim Vos, Nathalie Maes, Jean Luc Canivet, Renaud Louis, Paul Meunier, Julien Guiot

**Affiliations:** 1 Department of Radiology, University Hospital of Liège, 4000 Liège, Belgium; colin.desir@chuliege.be (C.D.); paul.meunier@chuliege.be (P.M.); 2 Department of Medico-Economic Information, University Hospital of Liège, 4000 Liège, Belgium; mthys@chuliege.be; 3 Department of Pneumology, University Hospital of Liège, 4000 Liège, Belgium; monique.henket@chuliege.be (M.H.); an.frix@chuliege.be (A.-N.F.); benoit.ernst@uliege.be (B.E.); r.louis@uliege.be (R.L.); j.guiot@chuliege.be (J.G.); 4 Radiomics (Oncoradiomics SA), 4000 Liège, Belgium; sean.walsh@radiomics.bio (S.W.); mariaelena.occhipinti@radiomics.bio (M.O.); wim.vos@radiomics.bio (W.V.); 5 Biostatistics and Medico-Economic Information Department, University Hospital of Liège, 4000 Liège, Belgium; nmaes@chuliege.be; 6 Department of Intensive Unit Care, University Hospital of Liège, 4000 Liège, Belgium; jean-luc.canivet@chuliege.be

**Keywords:** COVID-19, screening, HRCT

## Abstract

During the COVID-19 pandemic induced by the SARS-CoV-2, numerous chest scans were carried out in order to establish the diagnosis, quantify the extension of lesions but also identify the occurrence of potential pulmonary embolisms. In this perspective, the performed chest scans provided a varied database for a retrospective analysis of non-COVID-19 chest pathologies discovered de novo. The fortuitous discovery of de novo non-COVID-19 lesions was generally not detected by the automated systems for COVID-19 pneumonia developed in parallel during the pandemic and was thus identified on chest CT by the radiologist. The objective is to use the study of the occurrence of non-COVID-19-related chest abnormalities (known and unknown) in a large cohort of patients having suffered from confirmed COVID-19 infection and statistically correlate the clinical data and the occurrence of these abnormalities in order to assess the potential of increased early detection of lesions/alterations. This study was performed on a group of 362 COVID-19-positive patients who were prescribed a CT scan in order to diagnose and predict COVID-19-associated lung disease. Statistical analysis using mean, standard deviation (SD) or median and interquartile range (IQR), logistic regression models and linear regression models were used for data analysis. Results were considered significant at the 5% critical level (*p* < 0.05). These de novo non-COVID-19 thoracic lesions detected on chest CT showed a significant prevalence in cardiovascular pathologies, with calcifying atheromatous anomalies approaching nearly 35.4% in patients over 65 years of age. The detection of non-COVID-19 pathologies was mostly already known, except for suspicious nodule, thyroid goiter and the ascending thoracic aortic aneurysm. The presence of vertebral compression or signs of pulmonary fibrosis has shown a significant impact on inpatient length of stay. The characteristics of the patients in this sample, both from a demographic and a tomodensitometric point of view on non-COVID-19 pathologies, influenced the length of hospital stay as well as the risk of intra-hospital death. This retrospective study showed that the potential importance of the detection of these non-COVID-19 lesions by the radiologist was essential in the management and the intra-hospital course of the patients.

## 1. Introduction

Since the end of December 2019, the COVID-19 pandemic has become a global health crisis [1,2,3]. Its diagnosis is most commonly based on RT-PCR [4,5]. In the early phase of the pandemic, healthcare had to deal with the inherent delay in diagnosis due to the processing time of the RT-PCR to confirm the diagnosis of suspicious cases. Facing a massive influx of suspected COVID-19 patients with limited availability of PCR diagnosis, clinicians took the opportunity of using chest imaging to help in the diagnostic approach. Indeed, patients infected with COVID-19 exhibit typical chest-CT lesions that can easily confirm the diagnostic suspicion.

The most frequent lesions on the positive COVID-19 chest CT showed good sensitivity for the diagnosis [6,7]. The main CT semiologies of COVID-19 pneumonia are bilateral ground-glass opacities located mainly in the subpleural and posterior areas. They are often associated with zones of focal condensation and crazy paving appearance in later forms. Therefore, CT scan has become a highly frequent test for screening and diagnosing patients having symptoms possibly inherent to COVID-19, such as dyspnea, polypnea and/or desaturation requiring hospital treatment (and also with a view to a preoperative assessment) [7,8]. Thus, thousands of chest CTs were performed at patients’ admissions, whether with a confirmed or unconfirmed diagnosis of COVID-19 pneumonia. It must be noted that the diagnosis of COVID-19 on chest CT has a low specificity and mimickers may exist [9]. These various examinations have also indirectly led to de novo discoveries of non-COVID-19 associated chest abnormalities.

The outbreak of the COVID-19 pandemic also allowed the development of multiple artificial intelligence (AI) projects in order to assist the diagnosis, particularly by automating the assessment of the evolution of the disease and the quantification of lesions. Many tools were developed to classify and specify the lung abnormalities and quantify their extension (CO-RADS—for Reporting and Data System—and COVID-RADS, for example) [10,11]. At the University Hospital of Liège, we mainly used the COVIA system [12], which analyzes the whole lung field at a tomodensitometric level. Many other systems (such as the one developed by Robovision) were also used or tested. The COVIA system helps to identify an infection due to COVID-19 with 89.7% accuracy (95% CI: 84.0–93.9%). These analysis systems are essentially based on the identification of sensitive but not very specific COVID-19 lesions. On the other hand, the limitations of such automated or semi-automated approaches highlight the role of the radiologist’s systematic diagnostic workup and his expertise in the detection and analysis of incidental occurrences of non-COVID-19 lesions.

In addition, the multiple chest CT performed in the context of screening for COVID-19 infection on a large population provides possibilities to retrospectively analyze the potential benefit of screening patients with chest CT. The value of screening for lung cancer by chest CT is already well established in patients with a history of smoking habits [13,14]. The benefits of annual screening for lung cancer by chest CT in a patient aged from 55 to 80 consuming at least 30 packs of cigarettes/year allow early diagnosis and better management [15]. The scanners performed as part of the COVID-19 screening provide a database for carrying out an analysis of unknown non-COVID-19 pathologies on a large population, which would thus reflect mass screening.

The aim of this article is to use the study of the occurrence of non-COVID-19-related chest abnormalities (de novo or known lesions) in a huge cohort of patients having suffered from confirmed COVID-19 infection and statistically correlate the clinical data and the occurrence of these abnormalities in order to assess the potential of increased early detection of lesions/alterations. Hypotheses are that a correlation exists between the clinical data, epidemiological data and these observed lesions, which would be more pronounced for de novo lesions. These correlations would then impact parameters such as length of stay or potential intra-hospital death.

## 2. Materials and Methods

We retrospectively studied a sample of adult patients (n = 362) admitted to the emergency department with proven COVID-19 infection between 2 March and 7 June 2020 and who benefited from one or more chest CT scans at the University Hospital of Liège.

The protocol was approved by the ethics committee of the University Hospital of Liège (Belgian number: B707201422832; ref:2020/127).

No written informed consent from all subjects (patients) is applicable as this study uses anonymous data.

### 2.1. Computerized Tomography Study

Images were retrospectively reviewed by P.C. (medicine resident with 4 years of experience in radiology). The pre-existing database has been selected with a PCR proving patient infection with COVID-19. We then quantified and described any identifiable intrathoracic lesions (see Table 1). Chest CTs were performed using a CT scanner (Siemens Edge Plus, GE Revolution CT, GE Brightspeed) available at the University Hospital of Liège [16,17]. Most of these were carried out according to a standard chest high-resolution computed tomography (HRCT) protocol with spiral volume acquisition in spontaneous contrast, thin sections and multiplanar reconstructions. The main scanning parameters were: tube voltage, 120 kVp; automatic tube current modulation; pitch, 0.99–1 mm; matrix, 512 × 512; slice thickness, 1 mm; and field of view, 31.6 cm. All images were then reconstructed with a slice thickness of 1.250–5 mm with the same increment. Other scans include angio-scans, low-dose thoracic scans and, to a lesser extent, thoraco-abdominal scans (the study of which is limited to the thorax and the first abdominal cuts with the vertebral body of L2 as a reference) in the thoracic scanner database. We also compared the newly identified lesions with any other images or written reports available in the Picture archiving and communication system (PACS), which is provided by the enterprise Imaging “AGFA”. Pre-specified lesions which were already available in the medical imaging of the patient and the reports already available in PACS for this study were not considered de novo lesions. 

### 2.2. Statistical Analysis

Continuous variables were described using mean and standard deviation (SD) or median and interquartile range (IQR) as appropriate. Qualitative variables were presented with frequency tables (numbers and percentages).

Logistic regression models were used to analyze the impact of patients’ characteristics on the risk of presenting abnormalities on CT scans. Models were adjusted for age and gender. Results were presented using odd ratios (OR) and their 95% confidence intervals (95% CI) and “p” (*p*-value).

Linear regression models were used to analyze the impact of patients’ characteristics on the length of hospital stay. Models were adjusted for age and gender, and lengths of stay were log-transformed. Results were presented as regression coefficient estimation, standard error (SE) and “p”. Logistic regression models adjusted for age were used for risk of death at the hospital.

Results were considered significant at the 5% critical level (*p* < 0.05). Missing data were not replaced, and calculations were always performed on the maximum number of data available. Data analysis was carried out using SAS (version 9.4 for Windows). R (version 3.6.1) packages were used for the figures.

## 3. Results

We studied a group of 362 COVID-19-positive patients who performed a CT scan to diagnose and predict COVID-19-associated lung disease.

Patients’ descriptions are listed in Table 2. The average age is 65 years with a male/female ratio of 1.3, and 85% of patients were non-smokers. Associated comorbidities were chronic renal failure (11%), diabetic patients (39%) and arterial hypertension (58%). Among patients, 31% suffered a cardiovascular disease, and 18% suffered a chronic lung disease. The proportion of oncological patients was 13%. The median length of stay was ten days for 91% of the sample hospitalized for COVID-19, and 22% of the cohort required a passage to intensive care, while 5.1% (17/330) faced intra-hospital death.

In our cohort, we identified that 76% (280/362) of the population displayed non-COVID-19-related chest CT abnormalities. A total of 572 abnormalities were detected, with a proportion of 61% newly identified lesions, and 60.5% of the population was presenting new incidental lesions.

Length of hospital stay was increased in older (Coef. ± SE: 0.0084 ± 0.0033; “p” = 0.010) patients and in patients exhibiting obesity (Coef. ± SE: 0.46 ± 0.12; “p” = 0.0001). Co-morbidities such as diabetes (Coef. ± SE: 0.31 ± 0.10; “p” = 0.0017), hypertension (Coef. ± SE: 0.25 ± 0.11; “p” = 0.020), and chronic pulmonary disease (Coef. ± SE: 0.29 ± 0.13; “p” = 0.026) were also identified as risk factors of increased length of stay. In-hospital death’s risk was increased in smoking patients (OR (95% CI): 3.5 (1.2; 10); “p” = 0.021) and those known to suffer from active neoplasia (OR (95% CI): 3.6 (1.2; 10); “p” = 0.018). Of interest, we identified that patients exhibiting at least one pre-existing CT abnormality were at risk of increased in-hospital death by 2.8 (95% CI: (1.0; 7.9); “p” = 0.046) (See Table A3 and Table A4).

**Figure 1:**
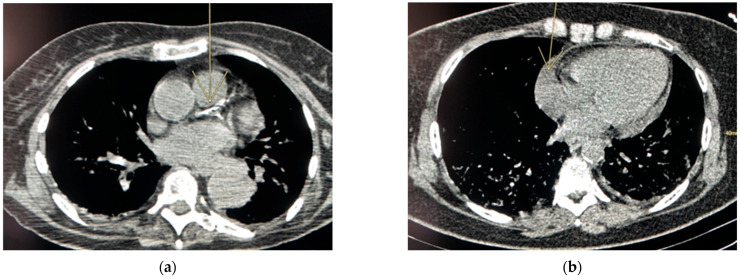
Calcified coronary atherosclerosis and pericardial effusion. (**a**) Chest CT of a 62-year-old woman performed in the context of suspected COVID-19 pneumonia with de novo discovery of calcifying atheromatosis. Coronary calcifications on left coronary artery ((left anterior descending artery and circumflex artery). (**b**) Chest CT of 58-year-old woman performed in the context of suspected COVID-19 pneumonia with novo discovery of a centimetric circumferential pericardial effusion.

The retrospective analysis of incidental features (see Table 3) showed that calcifying atheromatosis was the most frequent incidental de novo abnormality (example of chest CT in Figure 1a), with 35% represented in the sample with a percentage of more than half (60%) with already known calcifying coronary atheromatosis. The other cardiovascular parameters were less represented, with aneurysmal dilation of the ascending thoracic aorta in 9.7% and the presence of a significant pericardial effusion in 3.9% (example of chest CT in Figure 1b). Lung diseases were found in 19.4% of the population: 18% of the cohort showed COPD-associated lesions (mainly emphysematous lesions) and 1.4% signs of pulmonary fibrosis. Thyroid goiter was detected in 22% of the cohort. Of note, 63% (49/78) of this abnormality were not previously identified based on the medical file review.

Interestingly, we found that 8.8% of the patients were suffering from vertebral compression, whereas those lesions were not previously described in 3.3% of the total population.

**Figure 2:**
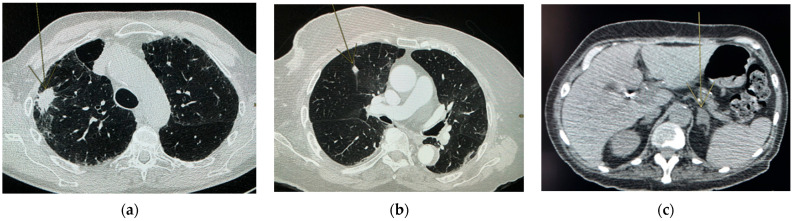
Suspicious mass and nodule. (**a**) Chest CT of an 81-year-old man performed in the context of suspected COVID-19 pneumonia with de novo discovery of suspicious mass. After biposing the lesion, the diagnosis is aspergilloma with usual interstitial pneumonia. Suspicious mass (> 3 cm) in the right upper lobe. (**b**) Chest CT of an 88-year-old woman performed in the context of suspected COVID-19 pneumonia, abdominal pain, nausea and vomiting with de novo discovery of suspicious nodule. Left lower lobe subpleural nodule. (**c**) Chest CT of a 62-year-old woman performed in the context of suspected COVID-19 pneumonia with de novo adrenal incidentaloma.

The presence of a nodule on the chest CT scan, regardless of its origin, was found in 26% of the sample. Incidental nodules were then identified in 18.8% of the total cohort (example of chest CT in Figure 2b,c). A newly identified suspicious mass or a suspicious lesion greater than 3 cm was found in 5% (example of chest CT in Figure 2a).

The proportion of known and unknown lesions is balanced in the sample except for a more marked difference for suspicious nodule, thyroid goiter and the thoracic aortic aneurysm, with a known proportion of lesions at 2.5% known versus 7.2% of unknown aneurysmal lesion.

### Risk Lesions

The probability of pre-existing or newly identified abnormalities increased with age (OR (95%CI):1.1 (1.1; 1.1); “p” < 0.0001) and is more frequently identified in men increasing the risk of presence by 1.8 (OR (95%CI): 1.8 (1.02; 3.2); “p” = 0.041) (see Table A1). The association with comorbidities also influences the likelihood of having abnormalities (3.5 x higher for smokers (95%CI: (1.2; 10)), and 9.4 x higher for patients with chronic kidney disease (95%CI: (1.2; 72))). There is no significant impact with respect to the other comorbidities on the probability of abnormalities on CT (*p* > 0.05).

The risk of having newly identified abnormalities on CT increases with the age of the patient (OR (95% CI): 1.04 (1.03; 1.1); “p” < 0.0001) and decreases with the presence of comorbidity, particularly in the context of arterial hypertension (OR (95% CI): 0.52 (0.3; 0.88); “p” = 0.014) and immunosuppression (OR (95% CI): 0.20 (0.073; 0.55); “p” = 0.0019) (see Table A2).

## 4. Discussion

In our study, we retrospectively identified that, among a cohort of 362 confirmed COVID-19 infected patients, incidental non-COVID-19-related chest lesions were mainly significant calcifying atheromatosis, suspicious nodule and thyroid goiter.

Age, male gender, having any COVID-19-associated comorbidity or active tobacco abuse were all specific risk factors for increased length of hospital stay (median (IQR) 10 days (6; 20)) or in-hospital death (5.1% of the hospitalized patients). Of interest, we showed that patients exhibiting at least one pre-existing CT abnormality were at increased risk of in-hospital death (risk multiplied by 2.8 (95% CI: (1.0; 7.9); “*p*” = 0.046)).

We identified that 60.5% of the population was presenting new incidental lesions. Of note, those were mainly calcified coronary disease, suspicious nodule and thyroid goiter. In our cohort, we found that 19% of patients were presenting incidental suspected lung nodule, which is in line with previous large screening cohort studies [27,28]. Based on the general recommendations of lung cancer screening, we also identified a subgroup of patients who were at higher risk of developing non-COVID-19 thoracic abnormalities. This population is characterized by older age, male gender and smoking status. In this context, a retrospective re-assessment of lung abnormalities after having performed a COVID-19 screening CT scan would be of interest and would increase the early detection of lesions/alterations.

Cardiovascular and pulmonary diseases have become major causes of death worldwide. According to the World Health Organization, cardiovascular diseases account for a major part with 17.5 million deaths, followed by 8.2 million for oncological and pulmonary pathologies [29]. Interestingly, the most frequent incidental lesion in our study is calcifying coronary atheromatosis, which, therefore, can have a significant impact on the patient outcome. Our data are in line with previous studies, identifying a global prevalence of calcifying coronary atheromatosis approaching 40% in a population of 60-year-old women [30], ranging up to 75% in patients over 70 in this study. With respect to a previous study [31], risk factors associated with severe COVID-19 infection are known to be mainly cardiovascular conditions, in opposition to previous respiratory chronic lung diseases [32,33].

In our cohort, the probability of encountering a thoracic lesion increases with age. The likelihood of developing calcifying atheromatosis, COPD signs or suspicious thoracic mass increases with age. Patients with pre-existing respiratory or cardiac comorbidities did frequently benefit from previous thoracic explorations leading to dedicated systematic medical and radiological follow-up [34]. This could potentially explain why we found in our study more incidental lesions in younger patients because they are more likely to have fewer comorbidities and, therefore, less routine medical follow-up. Patients with smoking history exhibit a four times higher risk of having lung lesions, whereas chronic renal failure provides nine times increased risk.

Similar to previous studies [35,36], our data show that length of hospital stay (LOS) in COVID-19-positive patients is influenced by many comorbid factors such as renal failure and hypertension. Age and comorbidities were found to be strong predictors of hospital admission and, to a lesser extent, of critical illness and mortality in people with COVID-19 [37]. Incidental identification of non-COVID-19-related thoracic lesion also appears to be an intrinsic risk factor for increased hospital LOS. It underlies the impact on potential comorbidities in severe COVID-19 patients. We thus identified that pre-existing signs of pulmonary fibrosis and vertebral compression were associated with an increased length of hospital stay. It is therefore essential to be able to better identify those comorbid lesions in order to propose a holistic approach to patients who are at higher risk of experiencing severe COVID-19 disease. However, our study did not show that a non-COVID-19 thoracic abnormality influences intensive care unit LOS. Unsurprisingly, the impact of in-hospital death is also correlated with comorbidities.

The comorbidities association such as a smoking or oncological history has shown to provide an increased risk of in-hospital death. In the study of Dai and al., patients with cancer (notably hematologic cancer, lung cancer, or metastatic cancer (stage IV)) appear more vulnerable to SARS-CoV-2 [38]. In addition, in our study, the mortality is increased by 2.8 times in patients with at least one abnormality identified with a CT scan. The presence of an incidental abnormality alone increasing the risk of in-hospital death should be subjected to multivariate analysis. Recent studies on COVID-19 showed that hypercholesterolemia, diabetes or COPD were associated with worse clinical outcomes, including COVID-19-associated mortality [37,38,39,40].

Taking into consideration the need for rapid CT-scan evaluation in the burden of the COVID-19 pandemic, it is nevertheless important to carefully evaluate non-COVID-19-related thoracic abnormalities as they are potentially influencing patient outcomes that can be overcome with artificial intelligence models.

### Limitation

In this study, we join the data at the global level regarding the importance of certain non-COVID-19 pathologies in the general population. However, the selection bias inherent in the positive COVID-19 patients selected in the study must be considered. We have shown that cardiovascular pathologies such as calcifying coronary atheromatosis were one of the most frequent pathologies in patients over 62 years of age, as found in the data in the literature, but the real background of this study is the impact of these non-COVID-19 chest lesions on patient management. This study showed that these lesions could represent unrecognized comorbidities with a significant impact on the patient. They could be integrated into a better assessment of the risk profile, especially since these lesions are generally not detected by artificial intelligence systems in the detection of COVID-19 pneumonia. The systematic approach of the radiologist, here more particularly, in the detection of non-COVID-19 lesions, thus plays a major role. The usefulness of the AI-based model in medical imaging is therefore complementary with the radiologist analysis in order to increase sensitivity and specificity of the diagnosis performance of chest CT.

## 5. Conclusions

This retrospective study showed that in the context of the pandemic, clinicians and radiologists had the chance to identify non-COVID-19-associated chest CT abnormalities. The high number of CT scan analyses performed also provides an opportunity to re-evaluate the occurrence of any thoracic abnormalities than can be incidentally identified in a general population. Correlations exist between clinical data, epidemiological data and these observed lesions, which can potentially be more pronounced for de novo lesions. These correlations could be confirmed if the data from this study are included in a meta-analysis which can be used to improve general patient care.

## Figures and Tables

**Table 1:** Chest CT features.

	Description
Nodule and mass	-Mass is defined as >3 cm (as the mass definition in lung CT [18])
	-Nodule of variable origin (pulmonary, lymphadenopathy, thyroid, adrenal, breast, others) (for example, in lung [18] or in adrenal [19])
Pulmonary diseases	-Signs of COPD (inflation, sign of bronchopathy, emphysema) [20]
	-Signs of pulmonary fibrosis (distribution of the attack, honeycomb, crosslinking, etc.) [21]
Cardiovascular diseases	-Signs of calcifying atheromatosis (coronary calcification, presence of stent)
	-Thoracic aortic aneurysm (diameter> 40 cm) [22]
	-Pericardial effusion (centimetric circumferential) [23]
Thyroid lesions	-Thyroid goiter (large thyroid with submerging goiter, presence of thyroid nodule) [24,25]
Spinal lesions	-Vertebral compression with loss of height of the vertebral body of a vertebra of the dorsal or lumbar column (L1 and L2) [26]

CT—computed tomography; COPD—chronic obstructive pulmonary disease.

**Table 2:** Patients’ characteristics (N = 362).

	n	Results
Age (years)	362	65.2 ± 15.8
Gender, male	362	204 (56.4)
Height (cm)	316	170 ± 10
Weight (kg)	312	79.5 ± 19.1
BMI (kg/m^2^)	294	27.6 ± 6.1
Smoking	330	
No		283 (85.8)
Stop > 6 months		29 (8.8)
Stop ≤ 6 months		2 (0.6)
Chronic		5 (1.5)
Occasional use		3 (0.9)
Yes		8 (2.4)
Chronic renal failure	282	32 (11.3)
Diabetes	352	138 (39.2)
High blood pressure	353	206 (58.4)
Obesity	307	83 (27.0)
Cardiovascular pathology	279	87 (31.2)
Chronic pulm. pathology	346	63 (18.2)
Immune suppression	279	22 (7.9)
Asthma	305	23 (7.5)
Oncologic patient	362	48 (13.3)
Hospitalization (COVID)	362	330 (91.2)
Length of stay (days)	330	10 (6; 20)
Intensive care unit	330	72 (21.8)
Deceased	362	40 (11.0)
At hospital		17
Not at hospital		23

Results are expressed as n (%), Mean ± SD or Median (IQR ); pulm.—pulmonary; BMI—body mass index.

**Table 3:** Abnormalities identified in CT scan (N = 362 patients).

	Absent	Present	Present and Known Based on Data Collected in the PACs	Present and Unknown Based on Data Collected in the PACs
Suspicious nodule	267 (73.8)	95 (26.2)	27 (7.4)	68 (18.8)
Suspicious mass	330 (91.1)	32 (8.9)	14 (3.9)	18 (5.0)
COPD sign	297 (82.0)	65 (18.0)	31 (8.6)	34 (9.4)
Sign of fibrosis	357 (98.6)	5 (1.4)	1 (0.3)	4 (1.1)
Calcified coronary atherosclerosis	146 (40.3)	216 (59.7)	88 (24.3)	128 (35.4)
Ascending aorta aneurysm	327 (90.3)	35 (9.7)	9 (2.5)	26 (7.2)
Pericardial effusion	348 (96.1)	14 (3.9)	4 (1.1)	10 (2.8)
Thyroid goiter	283 (78.4)	78 (21.6)	29 (8.0)	49 (13.6)
Vertebral collapse	330 (91.2)	32 (8.8)	20 (5.5)	12 (3.3)
Total number anomalies		572	223 (39.0)	349 (61.0)
Number anomalies/patient, mean ± SD		1.6 ± 1.3	0.62 ± 1.1	0.96 ± 1.0
0		82 (22.6)	251 (69.3)	143 (39.5)
1		114 (31.5)	51 (14.1)	132 (36.5)
2		84 (23.2)	26 (7.2)	58 (16.0)
3		50 (13.8)	19 (5.3)	19 (5.2)
4		21 (5.8)	12 (3.3)	9 (2.5)
5		10 (2.8)	3 (0.8)	1 (0.3)
6		1 (0.3)	0 (0.0)	0 (0.0)

PACs—other images or written reports available in the Picture archiving and communication system (PACS); COPD—chronic obstructive pulmonary disease; SD—standard deviation.

## Data Availability

Not applicable.

## Abbreviations

LOS: length of hospital stay; pulm.: pulmonary; patho.: pathology; CT: computed tomography; HRCT: high-resolution computed tomography; COVID-19: coronavirus disease 2019; SARS-CoV-2: severe acute respiratory syndrome coronavirus; RT-PCR: reverse-transcription polymerase chain reaction; CI: confidence interval; SD: standard deviation; IQR: interquartile range; SE: standard error; OR: odd ratios; “p”: p-value; PACs: Picture Archiving and Communication System; COPD: chronic obstructive pulmonary disease; AI: artificial intelligence.

## Appendix A

**Table A1:** Patients’ description in function of the presence of pre-existing or newly identified anomalies on CT scan (N = 362).

	0 Anomalies (N = 82)		≥1 Anomalies (N = 280)		Comparison
	N Non Missing	n (%) or Mean ± SD	N Non Missing	n (%) or Mean ± SD	Logistic Regression Adjusted for Age and Gender OR (95%CI), *p*-Value
Age (years)	82	52.3 ± 14.9	280	69.0 ± 14.0	1.1 (1.1; 1.1), <0.0001
Gender, male	82	41 (50.0)	280	163 (58.2)	1.8 (1.02; 3.2), 0.041
BMI (kg/m^2^)	65	30.0 ± 7.0	229	27.0 ± 5.7	0.96 (0.91; 1.003), 0.067
Smoking (including stopped)	74	5 (6.8)	256	42 (16.4)	3.5 (1.2; 10), 0.025
Chronic renal failure	76	1 (1.3)	206	31 (15.0)	9.4 (1.2; 72), 0.031
Diabetes	81	25 (30.9)	271	113 (41.7)	1.2 (0.65; 2.2), 0.59
High blood pressure	81	37 (45.7)	272	169 (62.1)	0.80 (0.43; 1.5), 0.50
Obesity	69	24 (34.8)	238	59 (24.8)	0.68 (0.35; 1.3), 0.25
Cardiovascular pathology	76	11 (14.5)	203	76 (37.4)	1.7 (0.77; 3.7), 0.20
Chronic pulmonary pathology	81	10 (12.3)	265	53 (20.0)	1.6 (0.72; 3.5), 0.26
Immune suppression	76	9 (11.8)	203	13 (6.4)	0.38 (0.14; 1.03), 0.058
Asthma	72	7 (9.7)	233	16 (6.9)	0.79 (0.28; 2.2), 0.65
Oncologic patient	82	5 (6.1)	280	43 (15.4)	1.8 (0.64; 4.9), 0.27

SD—standard deviation; OR—odd ratios; CI—confidence interval; BMI—body mass index.

**Table A2:** Patients’ description in function of the presence of unknown anomalies on CT scan (N = 362).

	0 Unknown Anomalies (N = 143)		≥1 Unknown Anomalies (N = 219)		Comparison
	N Non Missing	n (%) or Mean ± SD	N Non Missing	n (%) or Mean ± SD	Logistic Regression Adjusted for Age and Gender OR (95%CI), *p*-Value
Age (years)	143	59.5 ± 16.9	219	68.9 ± 13.9	1.04 (1.03; 1.1), <0.0001
Gender, male	143	76 (53.1)	219	128 (58.4)	1.4 (0.91; 2.2), 0.12
BMI (kg/m^2^)	119	28.3 ± 6.6	175	27.2 ± 5.8	0.99 (0.95; 1.03), 0.59
Smoking (including stopped)	132	21 (15.9)	198	26 (13.3)	0.73 (0.38; 1.4), 0.36
Chronic renal failure	117	12 (10.3)	165	20 (12.1)	0.92 (0.42; 2.0), 0.84
Diabetes	139	58 (41.7)	213	80 (38.6)	0.70 (0.44; 1.1), 0.13
High blood pressure	140	83 (59.3)	213	123 (57.8)	0.52 (0.31; 0.88), 0.014
Obesity	124	36 (29.0)	183	47 (25.7)	0.97 (0.57; 1.7), 0.92
Cardiovascular pathology	115	26 (22.6)	164	61 (37.2)	1.2 (0.66; 2.2), 0.57
Chronic pulmonary pathology	138	19 (13.8)	208	44 (21.1)	1.7 (0.90; 3.1), 0.10
Immune suppression	115	16 (13.9)	164	6 (3.7)	0.20 (0.073; 0.55), 0.0019
Asthma	124	11 (8.9)	181	12 (6.6)	0.83 (0.34; 2.0), 0.67
Oncologic patient	143	20 (14.0)	219	28 (12.8)	0.69 (0.36; 1.3), 0.25

SD—standard deviation; OR—odd ratios; CI—confidence interval; BMI—body mass index.

**Table A3:** Impact of patients’ characteristics on the risk of death during hospital stay (N = 330 COVID-19-hospitalized patients).

	Alive at Hospital Discharge (N = 313)		Death during Hospital Stay (N = 17)		Comparison
	N Non Missing	n (%), Mean ± SD or Median(IQR)	N Non Missing	n (%), Mean ± SD or Median(IQR)	Logistic Regression Adjusted for Age OR (95%CI), *p*-Value
Age (years)	313	65.6 ± 15.0	17	71.5 ± 18.1	0.030 ± 0.018, 0.10
Gender, male	313	183 (58.5)	17	11 (64.7)	0.20 ± 0.27, 0.45
BMI (kg/m^2^)	261	27.8 ± 6.3	15	26.4 ± 5.5	0.97 (0.88; 1.1), 0.53
Smoking (including stopped)	288	40 (13.9)	17	6 (35.3)	3.5 (1.2; 10), 0.021
Chronic renal failure	245	31 (12.7)	13	1 (7.7)	-
Diabetes	306	125 (40.9)	17	8 (47.1)	1.3 (0.47; 3.4), 0.65
High blood pressure	306	182 (59.5)	17	12 (70.6)	1.3 (0.43; 3.9), 0.65
Obesity	270	79 (29.3)	15	2 (13.3)	-
Cardiovascular pathology	242	74 (30.6)	13	8 (61.5)	2.5 (0.74; 8.5), 0.14
Chronic pulmonary pathology	301	52 (17.3)	17	4 (23.5)	1.5 (0.46; 4.7), 0.52
Immune suppression	242	18 (7.4)	13	0 (0.0)	-
Asthma	269	22 (8.2)	13	0 (0.0)	-
Oncologic patient	313	38 (12.1)	17	6 (35.3)	3.6 (1.2; 10), 0.018
≥ 1 known anomalies on CT scan	313	95 (30.3)	17	10 (58.8)	2.8 (1.0; 7.9), 0.046
Suspicious nodule	313	25 (8.0)	17	2 (11.8)	-
Suspicious mass	313	12 (3.8)	17	2 (11.8)	-
COPD sign	313	24 (7.7)	17	4 (23.5)	3.2 (0.96; 10.8), 0.059
Sign of fibrosis	313	1 (0.3)	17	0 (0.0)	**-**
Calcified coronary	313	75 (24.0)	17	7 (41.2)	1.8 (0.63; 5.1), 0.27
Ascending aorta Aneurysm	313	7 (2.2)	17	1 (5.9)	**-**
Pericardial effusion	313	3 (1.0)	17	1 (5.9)	-
Thyroid goiter	312	24 (7.7)	17	4 (23.5)	3.2 (0.96; 11), 0.059
Vertebral collapse	313	19 (6.1)	17	0 (0.0)	**-**

SD—standard deviation; IQR—interquartile range; OR—odd ratios; CI—confidence interval; BMI—body mass index; CT—computed tomography; COPD—chronic obstructive pulmonary disease.

**Table A4:** Impact of patient’s characteristics on the length of stay (N = 330 COVID-19-hospitalized patients—linear regression on log-transformed length of stay adjusted for age and gender).

	Coef. ± SE	*p*-Value
Age (years)	0.0084 ± 0.0033	0.010
Gender, male	0.065 ± 0.10	0.51
BMI (kg/m^2^)	0.022 ± 0.010	0.014
Smoking (including stopped)	−0.23 ± 0.14	0.11
Chronic renal failure	0.25 ± 0.17	0.13
Diabetes	0.31 ± 0.10	0.0017
High blood pressure	0.25 ± 0.11	0.020
Obesity	0.46 ± 0.12	0.0001
Cardiovascular pathology	0.11 ± 0.12	0.39
Chronic pulmonary pathology	0.29 ± 0.13	0.026
Immune suppression	−0.0025 ± 0.21	0.99
Asthma	0.20 ± 0.20	0.33
Oncologic patient	−0.038 ± 0.14	0.79
≥1 known anomalies on CT scan	0.21 ± 0.11	0.054
Suspicious nodule	−0.022 ± 0.18	0.90
Suspicious mass	0.29 ± 0.24	0.23
COPD sign	0.057 ± 0.18	0.75
Sign of fibrosis	1.9 ± 0.88	0.034
Calcified coronary	0.11 ± 0.12	0.36
Ascending aorta aneurysm	−0.13 ± 0.32	0.69
Pericardial effusion	−0.19 ± 0.45	0.68
Thyroid goiter	0.089 ± 0.18	0.61
Vertebral collapse	0.48 ± 0.21	0.022

Coef—coefficient; SE—standard error; BMI—body mass index; CT—computed tomography.
